# Supplementary material for: Impact of four years of annually repeated indoor residual spraying (IRS) with Actellic 300CS on routinely reported malaria cases in an agricultural setting in Malawi
Source: PLOS Glob Public Health. 2024 Apr 24;4(4):e0002264. doi: 10.1371/journal.pgph.0002264 (PMC11042720; doi:10.1371/journal.pgph.0002264)
Supplement: S1 File — The S1 File contains additional information on methodological choices made throughout the research process, as well as additional data summaries to improve understanding of the dataset. List of included tables: S1 Table A. Characteristics of control clinics used for their selection.S1 Table B. Median malaria cases per year and per clinic.S1 Table C. Unadjusted incidence rates between IRS and non-IRS treated clinics per year. List of included figures: S1 Fig A. Weather patterns over time.S1 Fig B. Fitted harmonic regression line vs observed data points.S1 Fig C. Auto-Correlation Function (ACF) plot for 5 clinics.S1 Fig D. Partial Auto-Correlation Function (PACF) plot for 5 clinics.S1 Fig E. Illustration of IRS coding per clinic—not taking into account insecticidal degradation.S1 Fig F. Illustration of IRS coding per clinic—assuming linear degradation of the insecticide.S1 Fig G. Illustration of IRS coding per clinic—assuming exponential degradation of the insecticide. (PDF) [file pgph.0002264.s001.pdf]

Supplementary materials

Access to data and code via 10.5281/zenodo.10626879.

1 Selection of control villages

DHIS2 data was available for thirteen clinics within close proximity of Illovo. The inclusion criteria were:

- Type of health facility: mainly out-patient
- Distance to the boundary of one of the Illovo fields: <10km
- Completeness of data: >90%
- Altitude: <100m difference to Illovo fields' maximum elevation (102m)

Seven clinics met the minimum criteria. Clinics were then ranked on these criteria, and clinics with the lowest combined score were selected. Because the sizes of these catchment areas for these clinics are relatively large compared to Illovo clinics, three clinics were selected. The characteristics of these clinics are presented in Tab A.

Table A. Characteristics of control clinics used for their selection

Characteristics	Bereu	Chipwaila	Maperera
Missing datapoints	1	2	1
Diagnostic pathway			
IMCI (%)	21.44	10.1	6.19
In-patient (%)	0	0	0.01
Microscopy (%)	0	0	0
RDT (%)	78.56	89.9	93.81
Population (%)	21823	38452	13805
Distance to Nchalo field (km)	0.92	16.39	5.63
Distance to Alumenda field (km)	22.83	7.98	27.05

IMCI = Integrated Management of Childhood Illness, RDT = Rapid Diagnostic Test.

2 Data preparation

2.1 Malaria data

Tab B describes the median malaria cases per year and per clinic. The unadjusted incidences rates between IRS and non-IRS treated clinics were calculated for each year Tab C. Unadjusted IRRs ranged from 0.22-0.79 show the effect is relatively consistent throughout the four years, with the exception of 2015. The IRR of 0.22 in 2015 is most likely confounded by the steep drop in malaria incidence after weather circumstances stabilised after the extreme weather in early 2015.

Table B. Median malaria cases per year and per clinic.

Clinic	2015		2016		2017		2018		Overall	
Factory	17.74	(9.82-25.65)	10.21	(6.76-16.22)	7.42	(3.71-11.56)	6.26	(5.31-7.21)	7.71	(5.32-13.90)
Kalulu	21.81	(15.27-30.81)	12.33	(10.63-16.08)	9.14	(5.10-13.27)	7.78	(4.84-10.89)	11.42	(7.43-16.45)
Nkombedzi	62.10	(39.19-89.53)	20.67	(11.73-29.89)	11.45	(6.77-14.57)	6.33	(4.87-9.01)	11.57	(7.13-29.89)
Alumenda	53.79	(21.90-84.29)	12.73	(8.48-17.23)	20.83	(13.97-24.89)	9.26	(5.85-12.19)	15.57	(8.48-25.73)
Mwanza	53.92	(21.57-72.96)	16.11	(9.88-21.69)	21.00	(4.74-33.61)	14.28	(10.38-19.14)	16.92	(10.30-30.98)
Mangulenje	58.87	(32.08-72.32)	20.81	(16.67-28.74)	20.49	(16.42-28.30)	37.16	(20.53-48.74)	27.90	(18.35-46.62)
Lengwe	67.78	(35.38-141.32)	23.78	(11.53-32.96)	29.30	(6.98-76.22)	11.50	(7.95-30.43)	28.55	(8.90-62.10)
Intervention	34.90	(15.60-78.50)	15.70	(9.45-25.20)	13.30	(6.98-23.60)	9.74	(5.85-14.90)	14.38	(7.62-30.90)
Chipwaila	7.13	(3.33-8.87)	7.76	(6.49-13.16)	16.20	(9.32-25.47)	17.21	(9.01-20.99)	9.72	(6.79-18.54)
Bereu	29.18	(11.12-48.19)	14.61	(11.65-17.10)	18.96	(14.77-29.92)	18.41	(10.49-29.38)	16.30	(11.25-29.92)
Maperera	47.33	(29.49-60.75)	50.28	(36.24-60.42)	47.94	(44.42-60.49)	81.31	(69.25-95.60)	52.49	(44.42-72.66)
Control	17.10	(7.36-47.60)	15.50	(9.29-38.50)	25.50	(15.40-44.2)	25.90	(11.30-63.10)	21.51	(10.96-45.80)
Overall	30.54	(11.00-61.29)	15.60	(9.45-26.01)	16.21	(8.44-30.40)	11.05	(6.89-29.43)	16.21	(8.31-35.91)

Malaria cases are reported as monthly totals. For each clinic the median was taken over each year and is provided with the interquartile range in brackets. Totals for the Illovo / intervention clinics and the non-Illovo / control clinics are provided. Overall totals in the final column, calculated in similar fashion.

Table C. Unadjusted incidence rates between IRS and non-IRS treated clinics per year

		2015		2016		2017		2018	
Within IRS protective period*		No	Yes	No	Yes	No	Yes	No	Yes
IRS clinics (n = 7)	Cases	6419	118	939	1626	2131	847	1798	641
	Population	117962	21998	39906	105666	72089	790945	94006	62790
	N	71	13	22	62	40	44	50	34
	Incidence	54.42	5.36	23.53	15.39	29.56	10.71	19.13	10.21
non-IRS clinics (n = 3)	Cases	16806	-	16631	-	23373	-	27663	-
	Population	828636	-	867006	-	905376	-	932844	-
	N	36	-	36	-	36	-	36	-
	Incidence	20.28	-	19.18	-	25.82	-	29.65	-
Combined (n = 3)	Incidence	24.53	5.36	19.37	15.40	26.09	10.71	28.69	10.21
Unadjusted IRR			0.22		0.79		0.41		0.36

Incidence rates were calculated by first grouping observations in the data-set by intervention status (IRS/non-IRS), IRS protective window (yes/no, only for IRS clinics), and year. Cases represent sum of cases for each group, population is summed population for each group. Incidence for each group is presented as new cases per 1000 population per month, calculated as Cases / Population x 1000. N is the number of observations that shows how many data points have contributed to each calculation. Note that because of the variability in timing of IRS between clinics, clinics do not contribute the same number of months to each calculation. Population was rounded to a an integer for presentation in this table, but not for subsequent calculations. The reason rounding was necessary is due to the linear interpolation between years 2016 to 2019, for which Illovo census data was available, to calculate populations for the other years. *Protective period is defined at village level as a 6-month period, starting at a 1 month lag after the first month of IRS.

2.2 IRS data

IRS data was available for each village and every year within Illovo boundaries on start day, end day, number of targeted houses, number of sprayed houses. This had to be aggregated to match the malaria data, spatially (clinic) and temporally (monthly). Temporal aggregation was done by rounding the date of the last day of IRS application in a village to a monthly level. Spatial aggregation was done based on which clinic inhabitants of a settlement would most likely attend, after consulting the public health officer. Because IRS implementation dates varied greatly between villages, the total number of houses sprayed at a given month per village was calculated first, also assigning this number to the next 6 months. The village level numbers were then summed at clinic level and a cumulative percentage of coverage was calculated for each month. This resulted in a number between 0 and 1, according to the proportion of houses within a clinic catchment area that are assumed to be protected by IRS at a given month, under the assumption that IRS with Actellic 300CS is protective for 6 months. IRS was not assumed to immediately have an effect on malaria cases, taking into account the time from infection to presenting at the clinic. Therefore, different versions of the IRS variable with lags from zero to three months were created. An example of the IRS coding is provided in Fig E.

2.3 Weather station data

Weather data was available from 01-01-1999 to 31-01-2019. A great number of daily measured weather variables was available from the manual weather station: maximum temperature, minimum temperature, maximum humidity, minimum humidity, dry bulb temperature at 08:00, dry bulb temperature at 14:00, wet bulb temperature at 08:00, wet bulb temperature at 14:00, relative humidity at 08:00, temperature at 14:00, total rainfall, solar radiation, hours of sunlight, number of dips from a class A pan, evaporation, wind run, wind speed, soil temperature at 5cm, soil temperature at 20cm, soil temperature at 100cm. To avoid collinearity only a few representative variable for temperature and humidity were chosen based on completeness, data distribution, and correlation with the automatic weather station data. Variables considered for further modelling: maximum temperature, minimum temperature, dry bulb temperature at 08:00, total rainfall, relative humidity at 14:00. One month of data was missing for relative humidity at 14:00, this was imputed by using the imputeTS package in R using linear interpolation. Because seasonality was already modelled through harmonic regression, the choice was made to model weather variables as anomalies, to represent variation outside of the seasonal trend. Because the malaria data was at a monthly level, weather data was aggregated to monthly level first. For rainfall the total rainfall per month was taken, for temperature and humidity monthly averages were taken. The long-term monthly averages for each julian month of the year (for example the average monthly mean for January) were calculated over the full dataset starting from 1999. This variable was subtracted from the observed values to obtain anomalies (how much the value differs from what is expected for that month based on the climatology). Lag-times from zero to three months were calculated for each of these anomalies. Because weather station data from 2014 was available, this was included within the lagged variables as well. Fig A shows the observed weather data to provide an idea of on the ground circumstances. Periods of intense rainfall are observed in early 2015 and in 2017.

3 Model structure

3.1 Overdispersion

Initially a Poisson model was considered to model the malaria counts, fitted using the glmer function from the R package lme4. To check for overdispersion (variance > mean), the ratio between the sum of the squared Pearson residuals (spr) and the residual degrees of freedom (rdf) was calculated. A hypothesis test for overdispersion was based on the Chi-Square distribution. Both the ratio of spr/rdf and p-value were calculated for basic models. Models based on the Poisson distribution

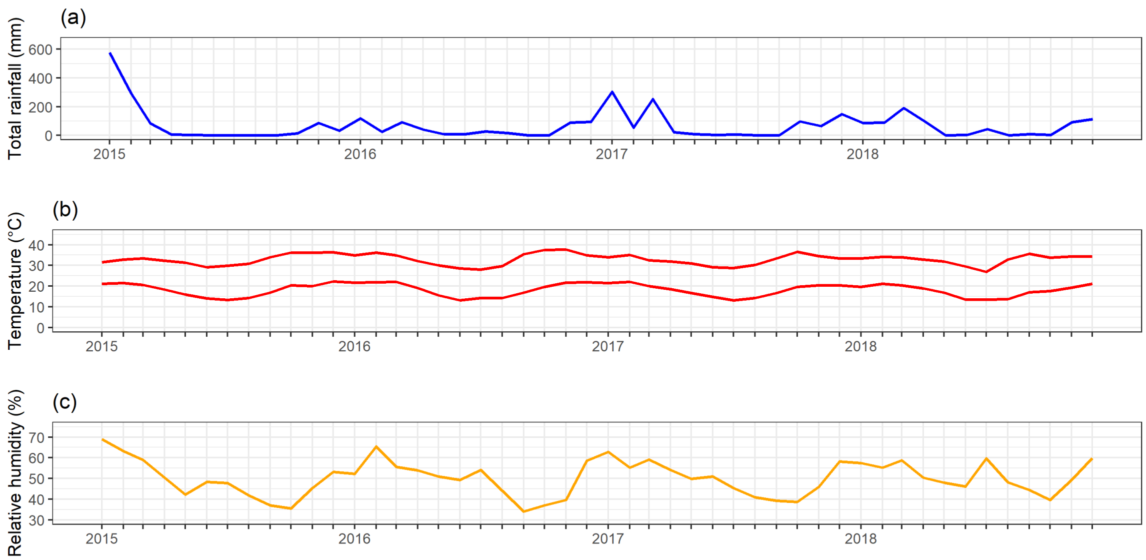

Fig A. Weather patterns over time. a) monthly total Rainfall (mm), b) Minimum and maximum temperature (°C), c) monthly mean humidity (%).

showed statistically significant ($p<0.001$) large ratios for over dispersion, therefore it was decided to continue further modelling steps with a negative binomial model using the glmer.nb function from the lme4 R package.

3.2 Random effects and offset

To account for unmeasured heterogeneity related to clinic catchment areas, a random intercept for clinic was added. An offset to account for differences due to population size was added to the model. When adding a random slope for time to a negative binomial model that included an offset for population size and a random effect, this reduced the AIC and was therefore adopted into the model structure.

4 Modelling seasonality

Although it is not an explicit goal to estimate the parameters for the harmonic regression component in this piece of work, if desired the the amplitude (A) and horizontal shift (θ) can be calculated:

$$A = \sqrt{\beta_1^2 + \beta_2^2} \tag{1}$$

$$\theta = arctan\left(\frac{\beta_1}{\beta_2}\right) * \frac{T}{2\pi} \tag{2}$$

The model formulation to produce Fig C:

$$\log(\lambda_{ct}) = \alpha + +\beta t + \gamma_1 * sin\left(\frac{2\pi t}{T}\right) + \gamma_2 * cos\left(\frac{2\pi t}{T}\right) \tag{3}$$

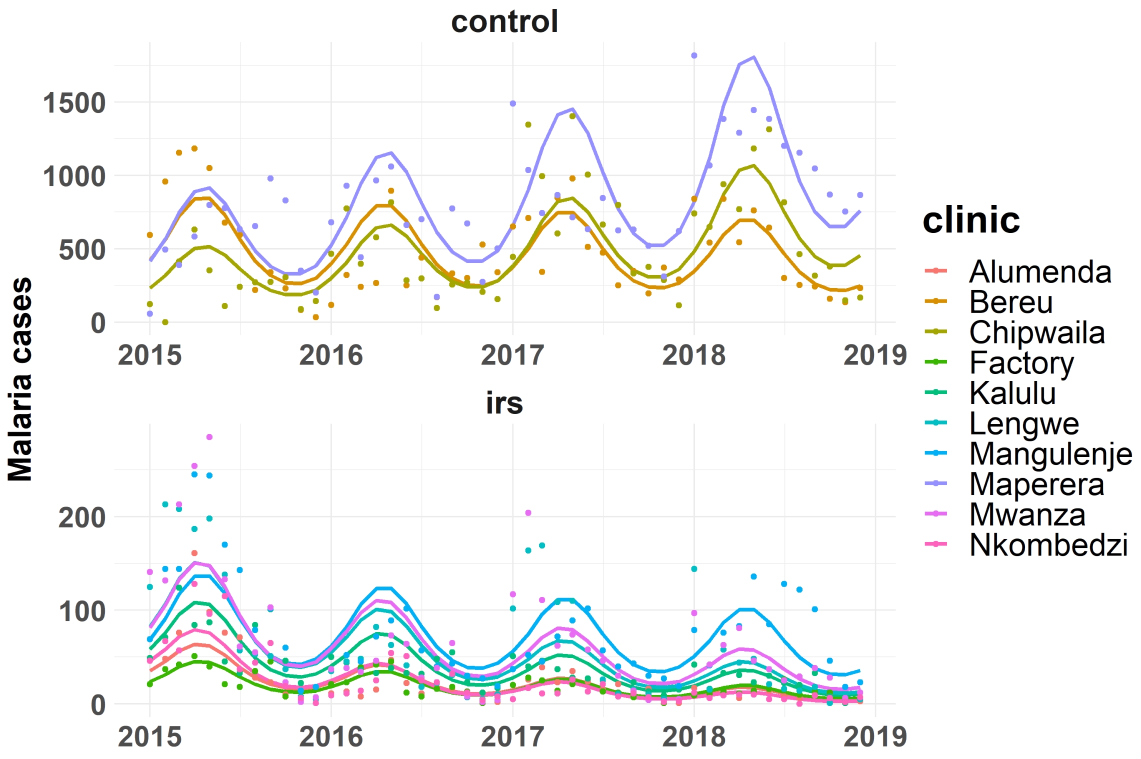

Fig B. Fitted harmonic regression line vs observed data points. Fitted harmonic regression lines and observed data points plotted for each clinic. Note that the top panel showing the control clinics has a differently scaled y-axis than the bottom panel with IRS clinics.

5 Final model results - untransformed coefficients

The model results without any transformations applied to the coefficients are presented in Tab D.

Table D. Coefficients of the final model

Covariate	Coefficient	SE	Z-value	P-value	95% CI	
Intercept	-3.5566	0.2391	-14.8719	0.0000	0.0144	0.0379
Time (days)	-0.0164	0.0073	-2.2540	0.0242	-4.0710	-3.0429
Sin-term*	-0.0023	0.0004	-5.1214	0.0000	-0.0322	-0.0007
Cos-term*	0.0042	0.0004	11.1795	0.0000	-0.0032	-0.0014
Rain anom. lag 3**	0.0841	0.0355	2.3676	0.0179	0.0035	0.0049
Relative humidity anom.***	-0.2933	0.0632	-4.6394	0.0000	0.0154	0.1548
Max temp anom. lag 1***	-0.4520	0.2470	-1.8299	0.0673	-0.4172	-0.1693
IRS lag 1	-0.6899	0.0867	-7.9597	0.0000	-0.9362	0.0320

Results of the final model. *As defined in section on modelling seasonality, **variable re-scaled by factor 100, ***variable rescaled by factor 10. SE = Standard Error. CI = Confidence Interval. Time is month of the study period (1-48). IRS is expressed as a proportion of coverage for a specific month and clinic (0-1). Anomalies calculated as difference between value for that month and 20-year average.

6 Temporal auto-correlation

To demonstrate the level of auto-correlation, ACF and PACF plots of the full dataset and of the residuals after fitting model 5 from the main text in Fig D and Fig E. The ACF shows significant temporal auto-correlation and the PACF demonstrates that the extent does not go beyond lag 1 after the exclusion of indirect correlations.

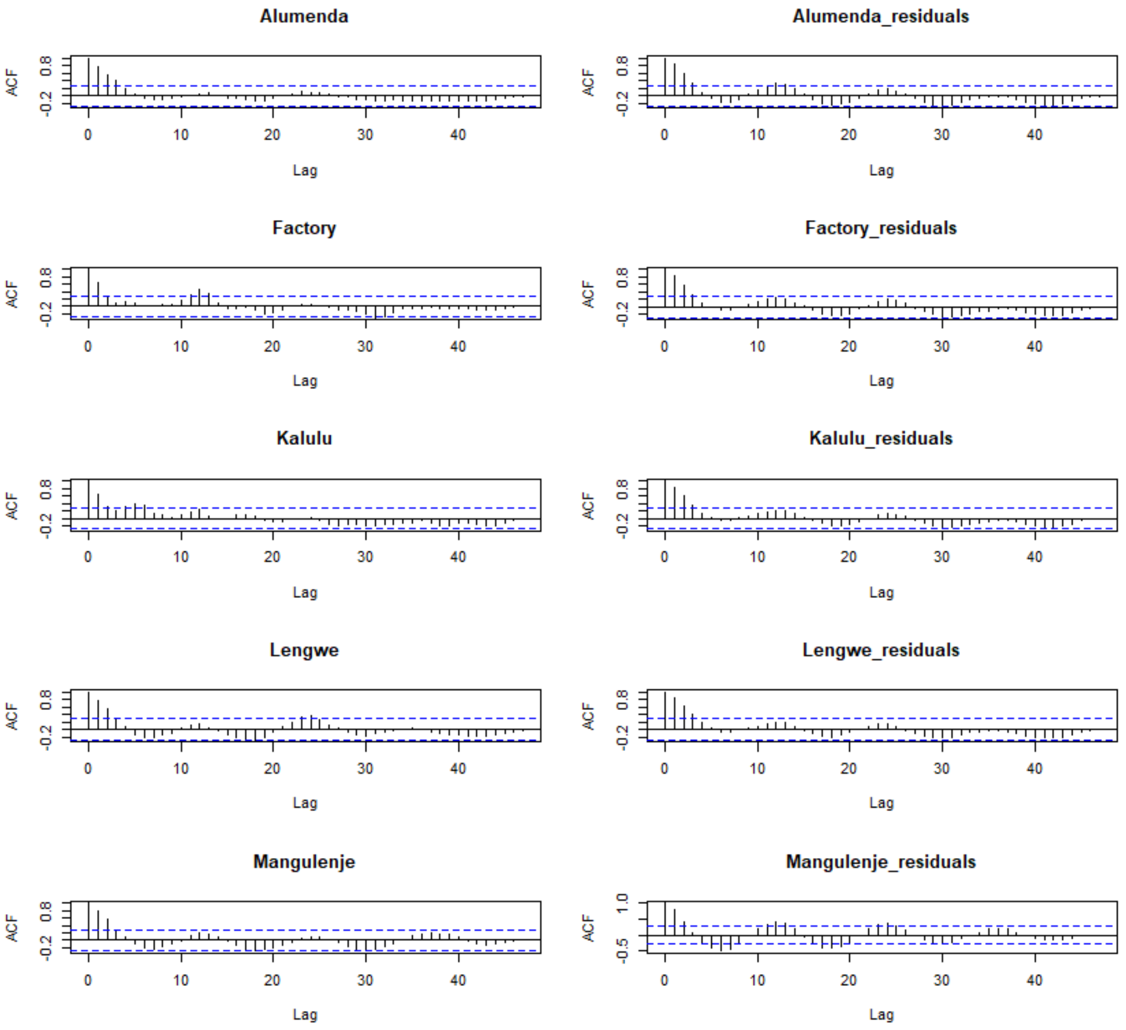

Fig C. Auto-Correlation Function (ACF) plot for 5 clinics.

7 IRS - insecticidal degradation and timing

Although it could not be fully be quantified in this analysis, some exploration has been done to visualise theoretical effects of IRS timing. The IRS coding scheme was adapted to assume three different types of insecticidal degradation: none, linear, exponential. In Fig E, Fig F, and Fig G, these different assumptions are visualised as shaded areas representing IRS coverage for the population over time, in contrast to the predicted malaria cases from the seasonal model without any additional covariates. It can be observed how both coverage in per village and different start dates when multiple villages report to one clinic (e.g. Mangulenje and Factory) changes the aggregation of

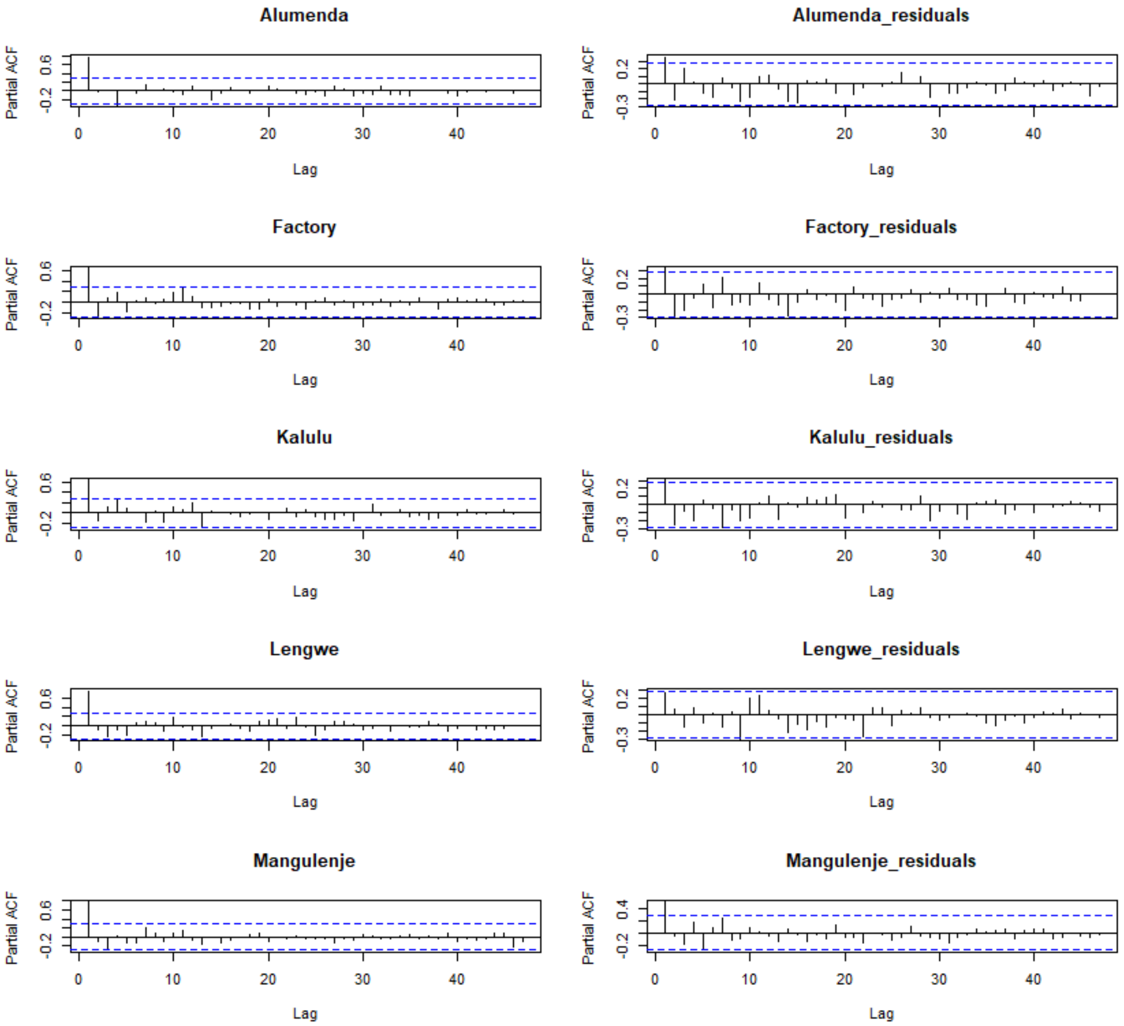

Fig D. Partial Auto-Correlation Function (PACF) plot for 5 clinics.

the IRS variable. It can be observed that for most clinics the IRS protective window does not fully cover the malaria season. An alternative interpretation of this is that after IRS stops being effective, malaria cases rebound. This stresses the importance of carefully planning the timing of IRS implementation across all villages in the target area. How insecticidal degradation is represented changes the proportion of the population without IRS protection during the malaria season as well. In this case no entomological data was available to support any form of a degradation curve and therefore the coding scheme of Fig E was used.

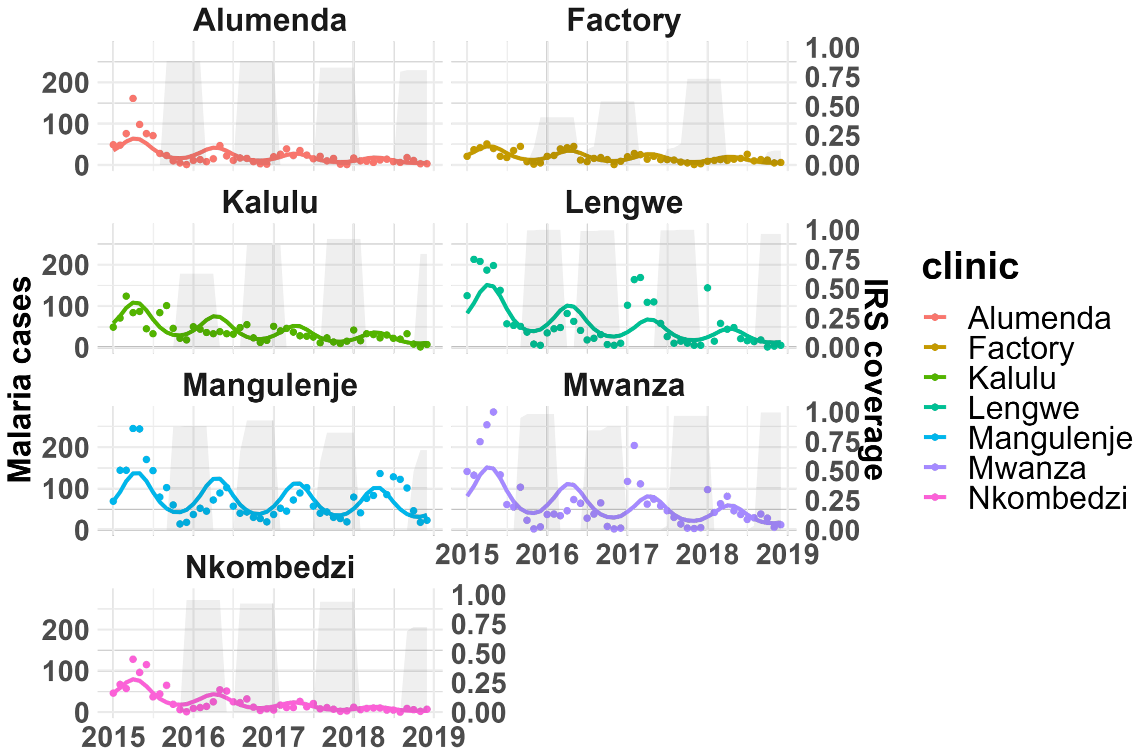

Fig E. Illustration of IRS coding per clinic - not taking into account insecticidal degradation The shaded areas represent the relative coverage for IRS for each clinic, based on households covered per clinic. Malaria cases are depicted as datapoints, with predicted cases based on the seasonal model without any additional covariates indicated by the line.

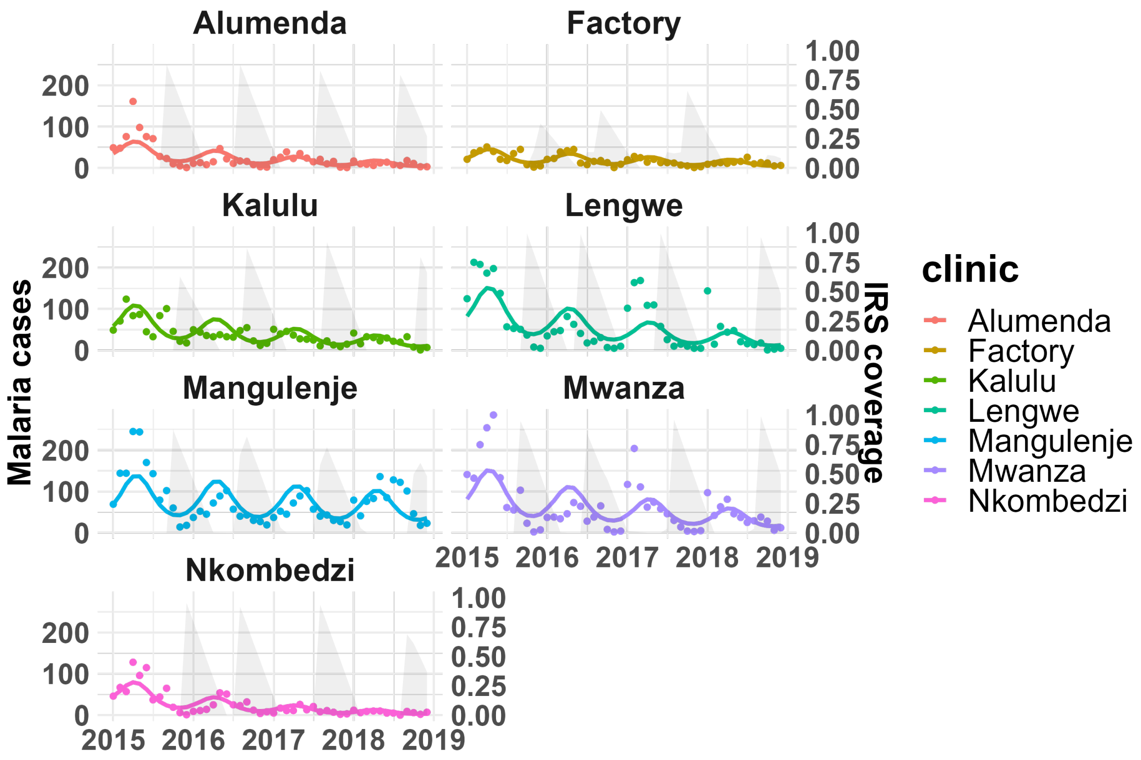

Fig F. Illustration of IRS coding per clinic - assuming linear degradation of the insecticide Describe.

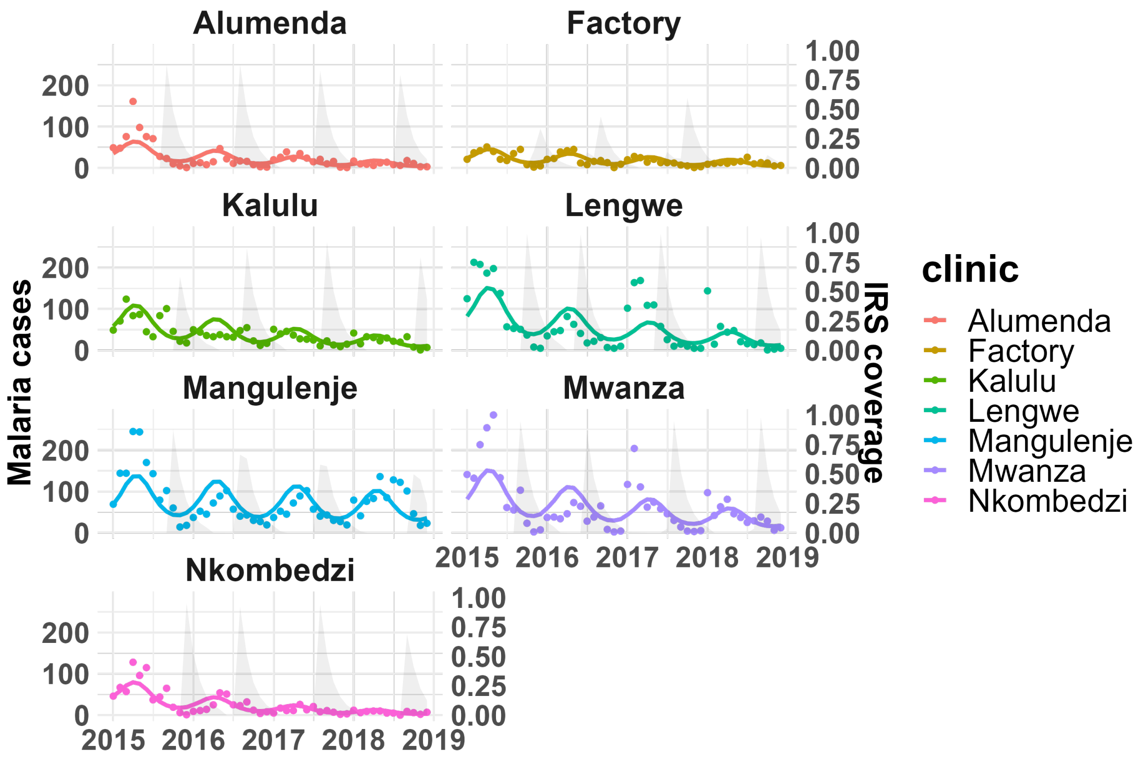

Fig G. Illustration of IRS coding per clinic - assuming exponential degradation of the insecticide Describe.
