# Supplementary material for: Impact of four years of annually repeated indoor residual spraying (IRS) with Actellic 300CS on routinely reported malaria cases in an agricultural setting in Malawi
Source: PLOS Glob Public Health. 2024 Apr 24;4(4):e0002264. doi: 10.1371/journal.pgph.0002264 (PMC11042720; doi:10.1371/journal.pgph.0002264)
Supplement: Attachment — Submitted filename: Response to Reviewers - 2.pdf [file pgph.0002264.s004.pdf]

06/02/2024

RE: Impact of four years of annually repeated indoor residual spraying (IRS) with Actellic 300CS on routinely reported malaria cases in an agricultural setting in Malawi

Reference: PGPH-D-23-01375R1- EMID:7bd2919c9cb38d6e

Dear reviewers,

Thank you for taking the time to go through my responses to the first round of review. Reviewer 1 indicated that the work was satisfactory and ready for publication. Reviewer 2 had a few additional comments, that have been addressed on the next page, replies to your comments are in red. I thank you for picking up on the mislabelled figures. I have provided clarification as requested and have updated Fig 3 to include prediction intervals as suggested.

Thank you for seeing the relevance of this article within the field of global health.

Sincerely,

Remy Hoek Spaans

Response to Reviewers

I am somewhat confused by the discussion of the results of the modelling. The random intercepts and slopes for the clinics are noted to be unusually high for Lengwe and Nkombedzi. The authors state this is due to (lines 225-226) “unmeasured covariates varying at clinic level”. But are these intercepts simply capturing the relative burden of cases at t = 0? So, the interpretation is that Lengwe and Nkombedzi have higher cases relative to other clinics at this time? Both of these locations have relatively high and negative slopes indicating that, over the period studied, there has been reductions in cases. I am unsure how this demonstrates “unmeasured covariates”? I think simply rephrasing this would be a good idea. There will always be uncertainty that we are not capturing with models particularly when fitted to ecological data.

This was intended to emphasize that there were no village level explanatory variables available that could perhaps have absorbed some of village-level variation. I also wanted to highlight Lengwe and Nkombedzi because they showed a different pattern than the other Illovo clinics. The result was perhaps a strange combination of the two. Since the first point is already mentioned in the discussion, I will focus on the latter in the results section.

Original text: “After adjusting for seasonal effects and IRS, there are still unmeasured covariates that vary at the clinic level that are affecting malaria incidence, this is most pronounced for the Lengwe and Nkombedzi clinics. The decrease in time slope for Illovo clinics was generally greater compared to control clinics.”

Adapted text: “The decrease in time slope for Illovo clinics was generally greater compared to control clinics. Lengwe and Nkombedzi show a different pattern than the other Illovo clinics, with a higher random intercept and a negative time slope.”

I think there is an issue with Figures referenced in the text and attached. I think the Figure 4 legend reads as though it is describing Figure 2? Please carefully check throughout.

Thank you for pointing out this labelling issue. I have relabelled the figures and re-uploaded them.

The results refer to Fig 2 when I think you mean Fig 3? At line 223. But please see the note above as I think it is a mismatch with uploaded figures.

Thank you for pointing out this labelling issue. I have relabelled the figures and re-uploaded them.

Can you also show some uncertainty on the plots for Figure 3 (sampling from the posterior predictions) – these are very interesting and worth noting the uncertainty.

I have included prediction intervals in the graph. Since the methodology of this article is based on a frequentist modelling approach using the lme4 R package, rather than a Baysian method, I have used the bootMer function to bootstrap the intervals.

The methods section was updated to include: “Prediction intervals were calculated using the "bootMer" function from "lme4", using 1000 simulations, bootstrapping conditional on the random effects.”

The image caption was updated to include: “Prediction intervals were bootstrapped and indicate 2.5th and 97.5th percentiles from a 1000 simulations.”

The Zenodo repository was updated to a new version to include the extra lines of code (DOI:10.5281/zenodo.10626879).

Page 6 line 37, says "… 2 to 9 months or residual activity…", please correct to of

Corrected to "2 to 9 months of residual activity". Thank you for catching this typo.

It is noted that 3 clinics were selected for control states although others fitted the criteria. Out of interest, was it not possible to include all these to gain inference from more data? Even if the areas were capturing larger populations? There is no need to do anything in this publication but if the data are available, why not use them?

While a few more clinics met the **minimum** criteria, it was deemed more important to have good quality controls than a larger number of controls. Especially because most covariates were derived from the weather station located inside of the Illovo estate. I also wanted to make sure the environment/proximity to irrigated agriculture was not too different between control and Illovo clinics. I selected the control clinics with highest proximity to the Illovo fields, but not on the Eastern side where elevation was much higher. To aid selection a satellite background map in QGIS was used.
