# Supplementary material for: Impact of four years of annually repeated indoor residual spraying (IRS) with Actellic 300CS on routinely reported malaria cases in an agricultural setting in Malawi
Source: PLOS Glob Public Health. 2024 Apr 24;4(4):e0002264. doi: 10.1371/journal.pgph.0002264 (PMC11042720; doi:10.1371/journal.pgph.0002264)
Supplement: Attachment — Submitted filename: Review comments.docx [file pgph.0002264.s002.docx]

What are the main claims of the paper and how significant are they for the discipline?

This is an interesting study that considers the impact of spraying Actellic 300CS indoors in people’s homes on malaria incidence within a zone of privately managed sugar plantation. The study compares outcomes from 7 clinics serving the residential community within the plantation to data from 3 health facilities serving people living outside the plantation. The main finding reported is that the repeated IRS application does have benefit to the community of permanent residents in the plantation zone. It is a useful study particularly because little information is often available on privately managed spray campaigns, so this study adds insight to these types of operations.

Are the claims properly placed in the context of the previous literature? Have the authors treated the literature fairly?

The paper is well-contextualised given previous literature that considers research and policy recommendation efforts by the wider community.

**Scientific comments / concerns**

Are original data deposited in appropriate repositories? The original data and analysis code are not part of the original submission (summary data are noted in the supplement), please share these or share the repository for these if possible.

Data concerns:

The IRS cover is estimated for each village and then all villages that are serviced by a given clinic are aggregated to estimate a clinic level IRS cover metric. The cover is determined to be the proportion of sprayed houses of all targeted houses and set at this value from the final month of the spray team delivering the intervention to that village, for the next 6 months. What about the waning efficacy of the intervention? This can be quite different in different locations for various reasons that the authors note. Would this approach not over-estimate cover for patients particularly toward the later months of a campaign? I think the analysis would likely still find a positive benefit from IRS, but it would certainly be worth commenting on this in the discussion. This is especially important because the authors note that IRS could be sprayed closer to the transmission season in the discussion. Please comment more on the assumptions of this framework. Potentially, you could try incorporating some waning e.g. Opiyo et al 2022?

To expand on this a little bit, the incremental spraying period is relatively prolonged (May to November) – how did you account for the differential protective window across the population (see Opiyo et al 2022 for thoughts on this stepped protection)? Some households would likely be unprotected by November, when others are first gaining protection. The level of community protection would therefore be quite heterogeneous over the year. How did this incremental spraying change for 2015-2018? Could this have any effect on the transmission patterns observed?

The 2015 year was noted to be particularly unusually wet, and lost benefit from the IRS as pyrethroid-products were being used. Would this not skew the results to over-estimate the Actellic effect? The 2015 was scored to have IRS cover as 0 for all clinics, and as this is when the incidence in the plantation is higher than elsewhere, the following effects from the IRS cover > 0 variable would lead to higher IRR (I think?) Is this fair? Could you repeat the analysis starting later in that first year to avoid this potential problem? Or comment on this in the interpretations?

The methods indicate that “the previous month’s malaria cases were added to the model as an auto-regressive term to adjust for potential temporal variation.” Could this lead to over fitting if you also are including the temporal trend?

At the editors discretion, it might improve readability if the model selection section was shifted to methods and only the results from Model 5 were then presented in the results. There is some repetition in the results section (e.g. the start of paragraph after Table 2) that can be cut.

It would be useful to present the raw data and code or at least the coefficients and intercepts of the modelling process, at least in the supplement, allowing the reader to reproduce results shown in Table 3 (and look at the change in probability of cases rather than IRR which is sometimes a preferred method to translate the findings from these types of models e.g. see Andrew Gelman et al. Regression and other stories - book).

The major conclusion is that Actellic IRS provides protection. Please could you comment on integrated resistance management. What sort of monitoring is needed to ensure resistance does not develop to the organophosphate product – could you add some thoughts on when this product should be rotated?

The timing of the most recent ITN campaigns is likely important. The assumption is that it is equivalent for people within and outside the plantation.

**Minor**

*Introduction*

The statement in the introduction that “*Anopheles spp* are shifting towards more exophagic behaviour” is possibly a little strong. The referenced papers are indicative … perhaps soften and say “with evidence suggesting that *Anopheles spp* may be shifting …”

There is a reference for “adaptations to the house after spraying” (Opiyo et al 2022 DOI: 10.1371/journal.pgph.0000227)

*Results*

The mean monthly incidence for cases per 1000 at risk are slightly different in the main manuscript and supplement (only at the second decimal place) but perhaps best to align these.

In the results section, there is a sentence at lines 206-208 that does not make sense “For both treatment groups, the annual incidence pattern mimics the weather patterns from with a lag of 1-3 months”. Please rephrase. At this section, the results state the lag for the incidence from the weather patterns but this is hard to see in the supplement. Perhaps plot this directly and refer to the specific supplementary figure as needed.

The key paragraph for results is not presented until just before Fig 3 so perhaps rearrange to highlight this finding. There are some grammatical errors to fix that will help clarify the finding too.

Figure 4 is an important validation of the statistical model’s ability to capture the data trends. It would be interesting to comment more on when the model falls short in capturing the data trends and any explanations for this. Did you find small denominators for any of the mis-matched months for instance? Were there any village- or clinic- level events that could explain e.g. the unexpectedly high case incidence rates in Chipwalla in the first half of 2017?

*Supplement*

In the supplementary materials, the inclusion criteria indicate less than 10 km and less than 90% completeness of data – should these be greater than?

How are the median cases given in Table 2 calculated? Is it that the monthly cases are noted and then the median and interquartile ranges for the calendar year are calculated?

I think the presentation of cases, population and incidence in Table 3 is somewhat confusing. It looks like you have calculated incidence as:

(Cases x number of clinics)/(months x population) x 1000

Is this correct? If so, could you note that “Cases” is “average cases per clinic”? And that incidence is per 1,000 people?

I am somewhat confused on the IRS data (section 2.2). I understand from what is shown that, for each village, the study team had the start date, end date and number of targeted and sprayed houses i.e. the data for example could look like:

Started 1^st^ May, ended 4^th^ June, targeted 100 houses, sprayed 90 houses.

Would the team interpret this as “90% cover” in June? And that the residents of this village would be protected from June to November inclusive?

Then, if 3 villages attended 1 health clinic, is it correct that e.g.

Village A: Started 1^st^ May, ended 4^th^ June, targeted 100 houses, sprayed 90 houses. Popn 1000

Village B: Started 1^st^ July, ended 14^th^ Aug, targeted 100 houses, sprayed 80 houses. Popn 2000

Village C: Started 2st Aug, ended 24^th^ Aug, targeted 100 houses sprayed 85 houses. Popn 2000

The analysis assumed coverage to be:

June: 1000*0.9 + 2000*0 + 2000*0 = 900 / 5000 i.e. 18%

July: 1000*0.9 + 2000*0 + 2000*0 = 900 / 5000 i.e. 18%

Aug: 1000*0.9 + 2000*0.8 + 2000*0.85 = 4200 / 5000 i.e. 84%

Sep: 1000*0.9 + 2000*0.8 + 2000*0.85 = 4200 / 5000 i.e. 84%

Oct: 1000*0.9 + 2000*0.8 + 2000*0.85 = 4200 / 5000 i.e. 84%

Nov: 1000*0.9 + 2000*0.8 + 2000*0.85 = 4200 / 5000 i.e. 84%

Dec: 1000*0 + 2000*0.8 + 2000*0.85 = 3300 / 5000 i.e. 66%

If the above is what was done, no problem, as this is how I interpreted what was presented. If not, please could you clarify.

It would be helpful to add another axis to the right of Supplementary Figure 1 to indicate IRS cover.

There are some minor grammatical errors in the first paragraph of sections 2.2 and 4 – please correct these.

*Discussion*

Noted in the limitations is the potential likelihood that houses within the plantation are a higher standard to surrounding communities – could this contribute to the duration of the IRS protective window? Can you comment on this anymore within the paragraph on how this IRS product has performed elsewhere?

There is an interesting juxtaposition between the potential increased risk of bites within the plantation due to the irrigation versus the potential higher standard of care through smaller populations being serviced by each clinic and higher standard housing noted by the authors. Is any community engagement done to encourage the use of interventions – particularly ITNs?
