# Supplementary material for: Impact of four years of annually repeated indoor residual spraying (IRS) with Actellic 300CS on routinely reported malaria cases in an agricultural setting in Malawi
Source: PLOS Glob Public Health. 2024 Apr 24;4(4):e0002264. doi: 10.1371/journal.pgph.0002264 (PMC11042720; doi:10.1371/journal.pgph.0002264)
Supplement: Attachment — Submitted filename: Response to Reviewers.docx [file pgph.0002264.s003.docx]

13/10/2023

RE: Impact of four years of annually repeated indoor residual spraying (IRS) with Actellic 300CS on routinely reported malaria cases in an agricultural setting in Malawi

Reference: PGPH-D-23-01375 - EMID:e2eaa16aa28a5fbb

Dear Editor and reviewers,

Thank you for taking the time to review my manuscript and for providing me with feedback to improve the quality and interpretability of my work. Please find my responses to your comments below, in the hope that they sufficiently address any concerns you have raised. The first section addresses the editor’s comments. The second section only pertains to the comments made by reviewer 2, since reviewer 1 was satisfied with the original submission. Comments are represented in red, while my reply is in black.

Thank you for considering this manuscript for publication.

Sincerely,

Remy Hoek Spaans

**Editorial comments**

1. Please note that PLOS ONE has specific guidelines on code sharing for submissions in which author-generated code underpins the findings in the manuscript. In these cases, all author-generated code must be made available without restrictions upon publication of the work. Please review our guidelines at https://journals.plos.org/plosone/s/materials-and-software-sharing#loc-sharing-code and ensure that your code is shared in a way that follows best practice and facilitates reproducibility and reuse.

Code has been made available via the Zenodo repository (10.5281/zenodo.10003226)

2. Please amend your detailed Financial Disclosure statement. This is published with the article. It must therefore be completed in full sentences and contain the exact wording you wish to be published.

a. State what role the funders took in the study. If the funders had no role in your study, please state: “The funders had no role in study design, data collection and analysis, decision to publish, or preparation of the manuscript.”

b. If any authors received a salary from any of your funders, please state which authors and which funders.

If you did not receive any funding for this study, please simply state: “The authors received no specific funding for this work.”

This work was funded by the Medical Research Council through the Medical Research Council Doctoral Training Partnership (1965090) awarded to Remy Hoek Spaans through the Liverpool School of Tropical Medicine and Lancaster University. The funders had no role in study design, data collection and analysis, decision to publish, or preparation of the manuscript.

3. Please send a completed 'Competing Interests' statement, including any COIs declared by your co-authors. If you have no competing interests to declare, please state "The authors have declared that no competing interests exist". Otherwise please declare all competing interests beginning with twhe statement "I have read the journal's policy and the authors of this manuscript have the following competing interests:"

Dr. Albert Kumbwa is employed by Illovo Sugar as the main clinician at Illovo Sugar Nchalo. Peter Nasoni was employed as the public health officer at the time of data collection.

4. We ask that a manuscript source file is provided at Revision. Please upload your manuscript file as a .doc, .docx, .rtf or .tex.

The manuscript and supplementary source files are provided as a .tex file.

5. Some material included in your submission may be copyrighted. According to PLOS’s copyright policy, authors who use figures or other material (e.g., graphics, clipart, maps) from another author or copyright holder must demonstrate or obtain permission to publish this material under the Creative Commons Attribution 4.0 International (CC BY 4.0) License used by PLOS journals. Please closely review the details of PLOS’s copyright requirements here: PLOS Licenses and Copyright. If you need to request permissions from a copyright holder, you may use PLOS's Copyright Content Permission form. Please respond directly to this email or email the journal office and provide any known details concerning your material's license terms and permissions required for reuse, even if you have not yet obtained copyright permissions or are unsure of your material's copyright compatibility.

Potential Copyright Issues:

Fig 1: please (a) provide a direct link to the base layer of the map (i.e., the country or region border shape) and ensure this is also included in the figure legend; and (b) provide a link to the terms of use / license information for the base layer image or shapefile. We cannot publish proprietary or copyrighted maps (e.g. Google Maps, Mapquest) and the terms of use for your map base layer must be compatible with our CC-BY 4.0 license.

Note: if you created the map in a software program like R or ArcGIS, please locate and indicate the source of the basemap shapefile onto which data has been plotted.

If your map was obtained from a copyrighted source please amend the figure so that the base map used is from an openly available source. Alternatively, please provide explicit written permission from the copyright holder granting you the right to publish the material under our CC-BY 4.0 license.

Please note that the following CC BY licenses are compatible with PLOS license: CC BY 4.0, CC BY 2.0 and CC BY 3.0, meanwhile such licenses as CC BY-ND 3.0 and others are not compatible due to additional restrictions.

If you are unsure whether you can use a map or not, please do reach out and we will be able to help you. The following websites are good examples of where you can source open access or public domain maps:

* U.S. Geological Survey (USGS) - All maps are in the public domain. (http://www.usgs.gov)

* PlaniGlobe - All maps are published under a Creative Commons license so please cite “PlaniGlobe, http://www.planiglobe.com, CC BY 2.0” in the image credit after the caption. (http://www.planiglobe.com/?lang=enl)

* Natural Earth - All maps are public domain. (http://www.naturalearthdata.com/about/terms-of-use/)

The map was generated in QGIS. Instead of the previously used Google Satellite basemap, I have now downloaded Landsat imagery via the UGSG website and cited the source. Figure 1 has been recreated using the Landsat imagery.

Figure caption has been edited to:

“Fig 1. Study area. A) Location of the Illovo Nchalo Estate within Malawi.

Administrative boundaries courtesy of GADM database of Global Administrative Areas

and Regional Centre for Mapping of Resources for Development (RCMRD). B)

Agricultural fields of Illovo Sugar Nchalo. The seven Illovo health clinics that received

IRS are indicated by circles. The three control clinics that did not receive IRS are

indicated by triangles. Landsat-8 image courtesy of the U.S. Geological Survey,

Shapefiles of the M1 road and agricultural fields constructed by surveyors from Illovo

Sugar Malawi.”

Three references to the data sources have been added.

Data licenses can be checked via:

https://africaopendata.org/dataset/africa-water-bodies-2015

https://www.usgs.gov/faqs/are-there-any-restrictions-use-or-redistribution-landsat-data

https://gadm.org/data.html

An extra sentence in the methods section has been added: “An Illovo agricultural surveyor maps the area every year, for maps generated here, 2015 data was used.”

**Reviewer comments**

Are original data deposited in appropriate repositories? The original data and analysis code are not part of the original submission (summary data are noted in the supplement), please share these or share the repository for these if possible.

The original data is accessible via Zenodo (DOI: 10.5281/zenodo.10003226). Apologies if this was not clear from the submission. It was filled out during the submission process, but it did not occur to me that this was not accessible to reviewers. The code was not added previously but should be accessible now on Zenodo.

Data concerns:

The IRS cover is estimated for each village and then all villages that are serviced by a given clinic are aggregated to estimate a clinic level IRS cover metric. The cover is determined to be the proportion of sprayed houses of all targeted houses and set at this value from the final month of the spray team delivering the intervention to that village, for the next 6 months. What about the waning efficacy of the intervention? This can be quite different in different locations for various reasons that the authors note. Would this approach not over-estimate cover for patients particularly toward the later months of a campaign? I think the analysis would likely still find a positive benefit from IRS, but it would certainly be worth commenting on this in the discussion. This is especially important because the authors note that IRS could be sprayed closer to the transmission season in the discussion. Please comment more on the assumptions of this framework. Potentially, you could try incorporating some waning e.g. Opiyo et al 2022?

A valid point, especially considering your track record of publications on this topic. This is an issue I am aware of and that will be more thoroughly addressed in one of my future publications, motivated by the fact that I could not fully address it in this analysis. I did initially explore different methods of coding IRS for the publication currently under revision, which I have not mentioned in the original submission of the main article or supplementary, but which is reflected in the R script. Because this did not change the regression coefficient of impact of IRS much, I choose not to include it previously for clarity of the story. Another method of taking the waning effect into account would have been to include a variable for “time since IRS”, but this was not compatible with the limitations of the data. Due to mismatch in spatial resolution of the malaria (clinic) and IRS (village) data and the variation between the timing of IRS in each of the villages (reporting to same clinic), I could not construct a variable that would accurately represent the waning effect. As part of data exploration, I opted to transform the coding to wane in a linear and exponential fashion. As a visualisation it might have value to represent theoretical overlap between IRS coverage and malaria seasonality. Results from this exploration are now included in the supplementary for those interested and additional comments have been included in the discussion and the last results paragraph.

Edits in results section:

“The shaded areas that indicate a period of IRS protection, assumed to last 6 months from the start of spraying, often co-occur with periods of lower malaria cases for the Illovo clinics. This period of low incidence is followed by an increase of cases after the protective window of IRS coverage, which could explained by either the impact of IRS, the natural seasonal pattern, or a combination of both. The seasonal pattern for control clinics does not appear vastly different than that of IRS covered clinics. Considering these observations, the timing of IRS is sub-optimal should be considered. Visualisations of IRS coverage in relation to malaria seasonality are presented in Supporting information to facilitate theoretical discussion on timing and October representation of insecticide degradation over time. In Fig 3, similar to Fig 2, a difference in temporal slope can be observed; an increase for control clinics and a decline for most of the Illovo clinics. This is an indication that the long running malaria control programme at Illovo is having an effect.”

Edits in discussion section (I made it explicit where I cited the recommended paper here):

“Whilst a systematic review of hut trials shows that the probability of pirimiphos-methyl killing mosquitoes entering the house starts to decline after 6 months, there are not many studies looking at the time-span for which Actellic 300CS provides protection against malaria and which factors affect its effectiveness in the field [Opiyo 2022, 14]. Our study reports effective protection for an assumed period of 6 months, but finer-scale temporal data is needed to provide more insight. Field reports from PMI Malawi suggest a residual life of 2-5 months, much lower than effectiveness measured in experimental studies, which could be explained by environmental and housing factors influencing degradation of the insecticide, or IRS application procedures [24]. Additionally, the long implementation period of the IRS campaign could have resulted in different windows of protection between villages, potentially offering sub-optimal protection during the malaria season in some villages and better protection in others [Opiyo 2022]. How exactly this has affected the estimation of IRS effectiveness is difficult to quantify due to the necessary data aggregation from village to clinic level. While the results show that periods of lower malaria incidence coincide with periods of IRS, the natural seasonality pattern could also have contributed to the observed IRS effect estimate, although the inclusion of control clinics mitigate this concern somewhat.”

To expand on this a little bit, the incremental spraying period is relatively prolonged (May to November) – how did you account for the differential protective window across the population (see Opiyo et al 2022 for thoughts on this stepped protection)? Some households would likely be unprotected by November, when others are first gaining protection. The level of community protection would therefore be quite heterogeneous over the year. How did this incremental spraying change for 2015-2018? Could this have any effect on the transmission patterns observed?

I realised I only alluded to this in the introduction “For practical reasons and in anticipation of the malaria season, IRS campaigns are usually planned to finish close to the start of the wet season ”. Of course, ideally protection is highest during the malaria season, however this did not seem feasible in the context of a small private campaign, but can be expanded upon more in the discussion. To my own interpretation of the data and conversations with the public health officer, there was no clear pattern or reasoning behind the order in which villages were sprayed – other than perhaps logistical. Some additional comments have been included in the discussion and the last results paragraph to mention the timing of IRS rather than just the length of the protective period. They are incorporated in the same sections cited in response to the previous comment.

The methods indicate that “the previous month’s malaria cases were added to the model as an auto-regressive term to adjust for potential temporal variation.” Could this lead to over fitting if you also are including the temporal trend?

The auto-regressive term was considered, but it was not included in the final model. No further comments were made on this in the paper, because it is not relevant to the final model results and interpretation of the results. To prevent over-fitting cross validation was used in the evaluation of the models, which indeed evaluated the auto-regressive model (6) as worse than the final model (5).

See in the text: “The last constructed model, which includes a variable for malaria cases of the previous month to account for temporal auto-correlation, had a slightly improved AIC, but the RMSE and generalisability as assessed by CV-RMSE calculated from the model validation decreased.” – in the previous version I had omitted the “CV” by mistake.

The referenced sentence has been changed to: “The previous month's malaria cases were added to the model as an auto-regressive term to check if it would improve model fit in case there was any additional non-seasonal temporal variation”.

At the editors discretion, it might improve readability if the model selection section was shifted to methods and only the results from Model 5 were then presented in the results. There is some repetition in the results section (e.g. the start of paragraph after Table 2) that can be cut.

The first draft included this section within the methods and was moved after feedback from several individuals based on the argument that this is a result of the modelling process carried out based on methodological evaluation criteria. I prefer to keep it as is, but if the editor wishes it to be moved, I do not have strong objections.

The repetition you refer to is not in the text, it is the description of table 2. The formatting of text and table description look very similar in this document but will look different on the website I presume. The document was compiled from the PLOS Overleaf template.

It would be useful to present the raw data and code or at least the coefficients and intercepts of the modelling process, at least in the supplement, allowing the reader to reproduce results shown in Table 3 (and look at the change in probability of cases rather than IRR which is sometimes a preferred method to translate the findings from these types of models e.g. see Andrew Gelman et al. Regression and other stories book).

A table containing the coefficients and intercepts have been added to the supplementary material. The results can also be reproduced through the code available on Zenodo.

The major conclusion is that Actellic IRS provides protection. Please could you comment on integrated resistance management. What sort of monitoring is needed to ensure resistance does not develop to the organophosphate product – could you add some thoughts on when this product should be rotated?

The most recent data from Chikwawa District show that the major vectors remain fully susceptible to pirimiphos-methyl (<https://parasitesandvectors.biomedcentral.com/articles/10.1186/s13071-022-05299-3>) but as you rightly point out, there needs to be a proactive approach at preventing the build up of resistance. At a minimum, consistent monitoring (annual basis) using WHO standard tube or bottle assays should be conducted on the main vector species (*An. Funestus* and *An. Arabiensis* in Illovo) – before commencement of spraying to confirm insecticide susceptibility. Ideally, Illovo should consider using alternative classes of insecticide approved for IRS in Malawi (e.g. clothianidin, SumiShield) although there might be logistical and cost implications. A rotation once every 3 years or the use of mosaics would help delay any selection for resistant genotypes, especially in agricultural areas where pesticides with similar modes of action are used to protect crops. The use of insecticide mosaics has been adopted for sugar pest control at Illovo in the years since this study was conducted.

The following sentence with above mentioned citation was added in the introduction: “Vectors in the area remain fully susceptible to pirimiphos-methyl.”

The following sentence was added to the discussion: “To slow the emergence of resistant genotypes within mosquitoes and sugar pests, Illovo has been using a mosaic of insecticides with different modes of action since 2019.”

The timing of the most recent ITN campaigns is likely important. The assumption is that it is equivalent for people within and outside the plantation.

Yes, this is the assumption. Illovo was covered by government mass distributions, just as any other place in Malawi (years mentioned in introduction). No additional data was available on specific dates for ITN campaigns.

The following sentence was added to the discussion: “No household level data was available on bed net distributions and the analysis presented here is based on an assumption that level of ITN use is similar between Illovo and surrounding villages.”

Minor

Introduction

The statement in the introduction that “Anopheles spp are shifting towards more exophagic behaviour” is possibly a little strong. The referenced papers are indicative … perhaps soften and say “with evidence suggesting that Anopheles spp may be shifting …”

Suggestion accepted and corrected in introduction.

There is a reference for “adaptations to the house after spraying” (Opiyo et al 2022 DOI: 10.1371/journal.pgph.0000227)

Suggestion accepted and reference incorporated.

Results

The mean monthly incidence for cases per 1000 at risk are slightly different in the main manuscript and supplement (only at the second decimal place) but perhaps best to align these.

I changed the supplementary figure to match the manuscript, the reason behind it was a slight difference in code I used.

In the results section, there is a sentence at lines 206-208 that does not make sense “For both treatment groups, the annual incidence pattern mimics the weather patterns from with a lag of 1-3 months”. Please rephrase. At this section, the results state the lag for the incidence from the weather patterns but this is hard to see in the supplement. Perhaps plot this directly and refer to the specific supplementary figure as needed.

The sentence was removed to avoid confusion. All it was intended for was to point the reader to the figure that describes local conditions and the variables included in the model.

The key paragraph for results is not presented until just before Fig 3 so perhaps rearrange to highlight this finding. There are some grammatical errors to fix that will help clarify the finding too.

I have restructured the results and methods section and tried to reduce duplication where possible.

Figure 4 is an important validation of the statistical model’s ability to capture the data trends. It would be interesting to comment more on when the model falls short in capturing the data trends and any explanations for this. Did you find small denominators for any of the mis-matched months for instance? Were there any village- or clinic- level events that could explain e.g. the unexpectedly high case incidence rates in Chipwalla in the first half of 2017?

Having a small denominator is not much of an issue in this dataset, especially the control clinics serve quite a big population. See results section:

“The average population size over the four year study period residing within the combined catchment areas for IRS-treated clinics was 12,365, whilst the non-intervention clinics served a larger population of 73,622 on average”.

For 2017 specifically there was another small spike in rainfall (See rainfall patterns in supplementary), but you would expect this to affect all the clinics similarly. The locations of Chipwaila, Lengwe and Mwanza are not clustered together either (see map figure 1), which makes it unlikely that this is due to interaction between rainfall and soil absorption properties for example. Because this is passively surveillance data, there are the usual limitations of differences in access to healthcare, changes in catchment population through migration etc. which are unmeasured and difficult to pinpoint. What I can say is that the Illovo clinic’s population are more likely to be more similar to each other compared to the control clinics, because they are run by the same company and probably serve a population that is more stable over time – with the exception of a small percentage of seasonal workers. See methods section and discussion:

“The clinics provide free care to Illovo employees and their family members, registered as dependants. In fact, dependants make up the largest proportion of the Illovo population (80.0%), followed by employees (19.5%) with a permanent contract, and seasonal workers on a temporary contract (0.53%).”

“Other unmeasured variables that could have influenced malaria incidence levels in the region are behavioural factors such as outdoor sleeping and ITN use, but also the seasonal influx of workers during the harvesting season.”

Control clinics are also a bit further removed from the location of the weather station, although they are in reasonable range, which is part of the reasoning for the inclusion criterium "distance to the boundary of the Illovo fields (<10km)”.

The following sentence in the discussion has been expanded to:

“Smaller catchment areas at Illovo may mean access to care is better and managed in a similar way to each other opposed to the more varied catchment population and management of the control clinics, which is reflected in the model evaluation.”

*Discussion*

Noted in the limitations is the potential likelihood that houses within the plantation are a higher standard to surrounding communities – could this contribute to the duration of the IRS protective window? Can you comment on this anymore within the paragraph on how this IRS product has performed elsewhere?

Maybe, but I cannot be sure in what way. Initially we planned to go back and record more specific data, very similar to the Opiyo 2022 paper. However, due to COVID travel restrictions, this never happened. I therefore cannot say with confidence what wall types they used and if there were any modifications, or speculate on any behavioural factors that could influence the duration of the IRS protective window. As far as I am aware no papers have been published linking socio-economic status to likelihood of wall modification or time of IRS protection. No changes in the text have been made for this reason.

There is an interesting juxtaposition between the potential increased risk of bites within the plantation due to the irrigation versus the potential higher standard of care through smaller populations being serviced by each clinic and higher standard housing noted by the authors. Is any community engagement done to encourage the use of interventions – particularly ITNs?

Yes, a very interesting juxtaposition. As far as I am aware (based on conversations with the public health officer during a visit in early 2019), no specific community engagement was done relating to ITNs. Because the households are visited once a year for IRS, perhaps members of the spray team will engage in conversation with the household members, but this is just speculation. No changes in the text have been made because I don’t have data to support further claims.

Supplement

In the supplementary materials, the inclusion criteria indicate less than 10 km and less than 90% completeness of data – should these be greater than?

Yes, thank you for spotting that. Corrected.

How are the median cases given in Table 2 calculated? Is it that the monthly cases are noted and then the median and interquartile ranges for the calendar year are calculated?

Yes, that is exactly how they were calculated. I have added a little bit more text in the description of the table to hopefully make that more clear.

I think the presentation of cases, population and incidence in Table 3 is somewhat confusing. It looks like you have calculated incidence as:

(Cases x number of clinics)/(months x population) x 1000

Is this correct? If so, could you note that “Cases” is “average cases per clinic”? And that incidence is per 1,000 people?

Apologies for the lack of description. While it comes to the same answer the way I calculated it was:

Aggregate data per year, intervention status, IRS protective window status. The protective window changes per year.

- Calculate total number of cases over x number of months and 7 or 3 clinics
- Calculate total population over x number of months and 7 or 3 clinics
- Calculate observations (x number of months*number of clinics) within each category
- Calculate total cases per category / total population per category * 1000
- Report adjusted population (for either 3 or 7 clinics, because this is how the table is set up) by dividing by x number of observations * 7 or 3 clinics (to get an average population for that combination of time periods and clinics contributing to the population)

If you want to calculate it from the table it looks like this:

(total cases) / (adjusted population / number of villages * number of observations in the period) * 1000

For example: 6419 / (11630/7 * 71) * 1000

I understand that perhaps using “months” instead of observations is a bit confusing. And that using an adjusted population is more confusing than just reporting the total population for the number of observations. It was tricky to report because of the variation between timing of IRS between clinics.

To increase clarity, I have changed the table to now present the total population. I still want to report the number of observations, so that it is clear to the reader how many data points have contributed to the calculations.

I am somewhat confused on the IRS data (section 2.2). I understand from what is shown that, for each village, the study team had the start date, end date and number of targeted and sprayed houses i.e. the data for example could look like:

Started 1st May, ended 4th June, targeted 100 houses, sprayed 90 houses.

Would the team interpret this as “90% cover” in June? And that the residents of this village would be protected from June to November inclusive?

Yes

Then, if 3 villages attended 1 health clinic, is it correct that e.g.

Village A: Started 1st May, ended 4th June, targeted 100 houses, sprayed 90 houses. Popn 1000

Village B: Started 1st July, ended 14th Aug, targeted 100 houses, sprayed 80 houses. Popn 2000

Village C: Started 2st Aug, ended 24th Aug, targeted 100 houses sprayed 85 houses. Popn 2000

The analysis assumed coverage to be:

June: 1000*0.9 + 2000*0 + 2000*0 = 900 / 5000 i.e. 18%

July: 1000*0.9 + 2000*0 + 2000*0 = 900 / 5000 i.e. 18%

Aug: 1000*0.9 + 2000*0.8 + 2000*0.85 = 4200 / 5000 i.e. 84%

Sep: 1000*0.9 + 2000*0.8 + 2000*0.85 = 4200 / 5000 i.e. 84%

Oct: 1000*0.9 + 2000*0.8 + 2000*0.85 = 4200 / 5000 i.e. 84%

Nov: 1000*0.9 + 2000*0.8 + 2000*0.85 = 4200 / 5000 i.e. 84%

Dec: 1000*0 + 2000*0.8 + 2000*0.85 = 3300 / 5000 i.e. 66%

If the above is what was done, no problem, as this is how I interpreted what was presented. If not, please could you clarify.

Yes, this is correct.

It would be helpful to add another axis to the right of Supplementary Figure 1 to indicate IRS cover.

I changed the figures in response to your other comments. An example of the coding can now be seen as Figure 5. I have added the extra axis per request.

There are some minor grammatical errors in the first paragraph of sections 2.2 and 4 – please correct these.

I made some small edits in the referenced sections.
